# FAIRDOMHub: a repository and collaboration environment for sharing systems biology research

**DOI:** 10.1093/nar/gkw1032

**Published:** 2016-11-24

**Authors:** Katherine Wolstencroft, Olga Krebs, Jacky L. Snoep, Natalie J. Stanford, Finn Bacall, Martin Golebiewski, Rostyk Kuzyakiv, Quyen Nguyen, Stuart Owen, Stian Soiland-Reyes, Jakub Straszewski, David D. van Niekerk, Alan R. Williams, Lars Malmström, Bernd Rinn, Wolfgang Müller, Carole Goble

**Affiliations:** 1 Leiden Institute of Advanced Computer Science, Leiden University, Leiden, 2333 CA, Netherlands; 2 Heidelberg Institute for Theoretical Studies gGmbH, Schloss-Wolfsbrunnenweg 35, 69118, Heidelberg, Germany; 3 Department of Biochemistry, University of Stellenbosch, Private Bag X1 7602 Matieland, South Africa; 4 School of Computer Science, The University of Manchester, Kilburn Building, Oxford Road, Manchester, M13 9PL, UK; 5 University of Zurich, Winterthurerstrasse 190, Y12F64, 8057, Zurich, Switzerland; 6 ETH Zurich, Weinbergstrasse 11, 8092 Zurich, Switzerland

## Abstract

The FAIRDOMHub is a repository for publishing FAIR (Findable, Accessible, Interoperable and Reusable) Data, Operating procedures and Models (https://fairdomhub.org/) for the Systems Biology community. It is a web-accessible repository for storing and sharing systems biology research assets. It enables researchers to organize, share and publish data, models and protocols, interlink them in the context of the systems biology investigations that produced them, and to interrogate them via API interfaces. By using the FAIRDOMHub, researchers can achieve more effective exchange with geographically distributed collaborators during projects, ensure results are sustained and preserved and generate reproducible publications that adhere to the FAIR guiding principles of data stewardship.

## INTRODUCTION

The FAIRDOMHub is a repository for storing and sharing data, models, protocols and publications relating to systems biology research projects. The systems biology approach has an iterative cycle of experimental and modeling analyses. Experimental results inform mathematical model design and refinement, and modeling simulations direct further laboratory experiments. Data are highly heterogeneous and the relationships between multiple different data sets and mathematical models must be clearly maintained. The interlinking of the experimental data, standard operating procedures (SOPs) and models is essential for interpreting and understanding results. There are several well-established databases for mathematical models or types of experimental data (e.g. omics data and kinetics), but FAIRDOMHub combines data and models and provides services that enable the integration, interlinking and publishing of experimental and modeling results in the context of the overall systems biology experiment, from a project perspective.

The FAIRDOMHub (https://fairdomhub.org/) has been developed as a joint action between ERA-Net ERASysAPP (https://www.erasysapp.eu/), an EU-wide consortium of applied systems biology, the European Research Infrastructure and Infrastructure for Systems Biology in Europe (ISBE) (http://project.isbe.eu/). It builds on and combines the tools, services and expertise of the earlier SysMO-DB and Sybit data management projects that includes the SEEK platform and openBIS. To date, the FAIRDOMHub contains data, SOPs models, publications and other *research assets* from large research programmes and independent projects, including the SysMO consortium, ERASyAPP and Digital Salmon. It contains more than 1260 data files, 170 models and 200 SOPs, relating to 240 publications arising from some 40 projects. The underlying FAIRDOM software platform and related tools are also available for download and independent installation (1,2). Currently 22 independent instances have been deployed.

## MATERIALS AND METHODS

The FAIRDOMHub is a public service for users to upload and publish research assets. It serves as a collaboration tool before publication and as a supplementary materials resource to link data, models and other research assets to manuscripts after publication. Projects of all sizes can be registered; from large, multinational consortia to those undertaken by individual researchers. An example of a published (3) collection of data and models is the ‘Glucose metabolism in *Plasmodium falciparum* trophozoites’ investigation (https://fairdomhub.org/investigations/56) that has 3 Studies, 24 Assays, 16 Data files, 19 Models, 13 SOPs and 3 Publications. The Investigation describes the construction, analysis and validation of a model of glycolysis in the malaria parasite *P. falciparum*. The *Model Construction Study* describes the experimental kinetic characterization of each individual reaction. The *Model Validation Study* shows how the model performs and compares to experimental results for the system, and finally, the *Model Analysis Study* points at possible drug targets. This Investigation shows a typical systems biology experiment, with iterations between modelling and experimental data sets. The Investigations, Studies and Assays (ISA) structure on the FAIRDOMHub gives a complete listing of all experimental data and mathematical analyses and makes the model construction and validation transparent and reproducible.

Data sharing policies are upheld through assigned roles that consortia and projects manage themselves. Researchers remain in control of their own assets and can decide who to share them with, and when to publish them. To support data citation and credit each asset is assigned a persistent URL and retains a link to the person and project it came from (e.g. Model 138 is associated with the SARCHI project and two individual researchers from the project – https://fairdomhub.org/models/138). For publication, digital object identifiers (DOI) can be minted for individual assets or for a collection that forms part of an investigation (e.g. DOI: 10.15490/seek.1.investigation.56, which is referenced in (3)). This means that content can be directly cited by researchers. Assets relating to projects may also be stored in specialist public repositories such as SABIO-RK (4) for biochemical reactions and their kinetics and BioModels for models (5). Regardless of where they physically reside FAIRDOMHub organizes these assets in one place by linking out to other repositories. It thus aggregates content spread over distributed stores while retaining the context of the investigation; reusing public repositories and securely linking to project stores where data are too large or too sensitive to upload.

### Standards

The FAIRDOM platform provides tools and resources to help standards-compliance of uploaded assets to support, for example, the simulation of models and rich querying across the content. The Just Enough Results Model (JERM) is a Minimum Information Model created to describe the interrelations between assets in the FAIRDOMHub and the metadata fields required to describe them. It builds on, and combines, existing life science Minimum Information models (e.g. MIAPE), which aim to capture the least amount of information needed to understand and interpret an experiment, and the ISA structure that allows the aggregation of individual assays into related studies and investigations. JERM templates are defined and shared in spreadsheet format for different types of experimental data using the RightField annotation tool (6) (e.g. https://fairdomhub.org/data_files/1214). A collection of templates, developed in collaboration with users, are freely available through the Help Documents section. Although all model types are supported, the recommended standard for describing models is MIRIAM-compliant Systems Biology Markup Language (SBML) (7). SBML model components can be matched with data items from uploaded files to find new sources. Model versions can be compared using the BiVes (https://sems.uni-rostock.de/projects/bives/) plugin. SBML models are simulated through an integrated JWS Online plugin (8) (e.g. click the ‘Simulate Model on JWS’ button for https://fairdomhub.org/models/138). Simulation instructions can be stored in Simulation Experiment Description Markup Language (SED-ML) format (9) as part of a COMBINE Archive (10) that can be downloaded directly from the JWS simulator and is linked to the model analysis through JWS Online.

### Access, browsing and searching

**Figure 1.**
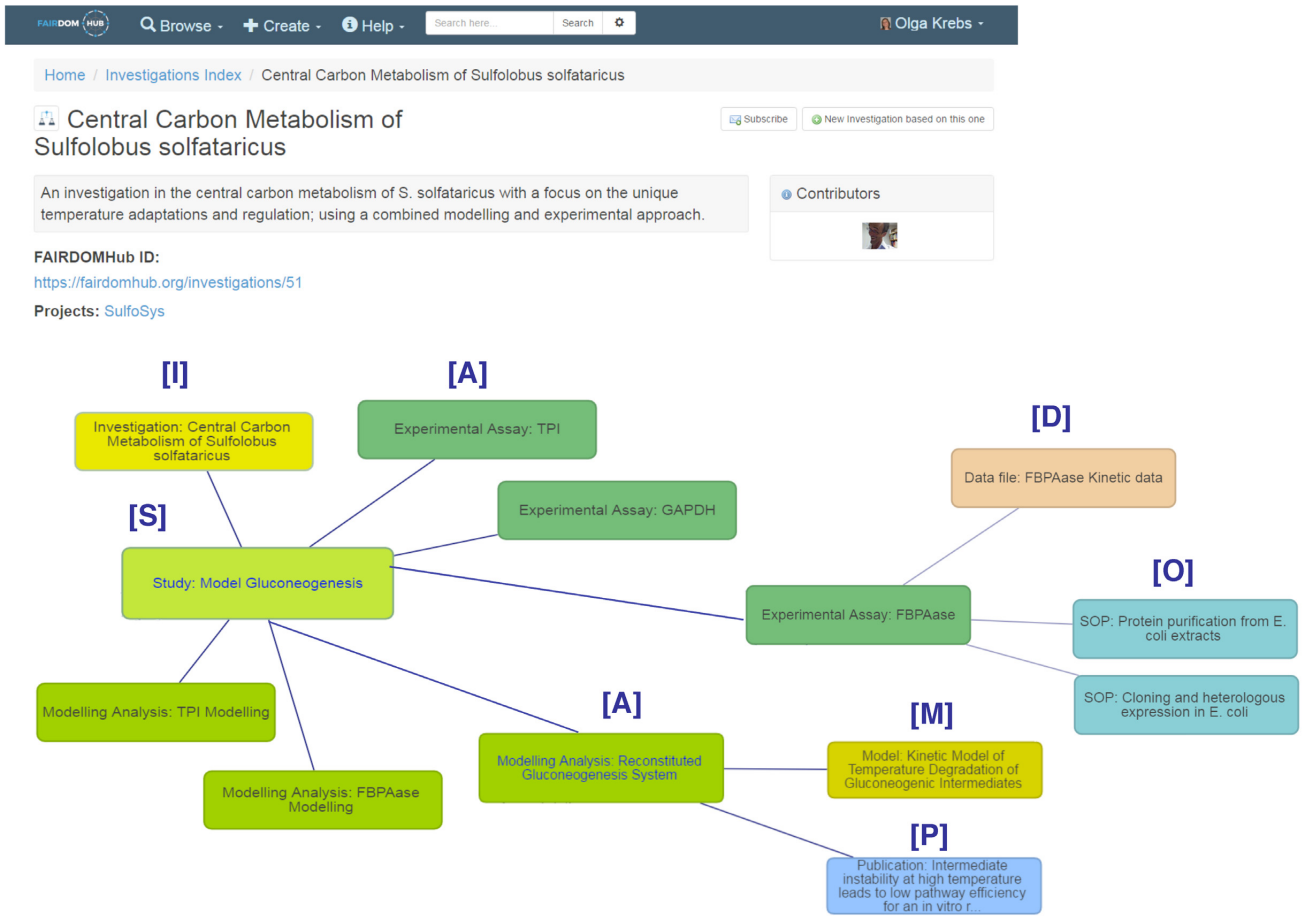
The FAIRDOMHub interface showing an Investigation into the Central Carbon Metabolism of *Sulfolobus solfataricus* (12) (https://fairdomhub.org/investigations/51) and the relationships between different assays in one of the constituent studies. The graphical view shows Investigations (**I**), Studies (**S**), Assays (**A**), Data (**D**), Models (**M**), Operations (or Standard Operating Procedures-**O**), and Publications (**P**). Assays are displayed in two groups; top right shows experimental assays, and bottom left shows modeling assays (analyses). This is a simpler investigation than that of (3) chosen for clarity on the printed page. In practice the interface is designed to be interactive so that users navigate through the graph. Our forthcoming folder-based view will provide an alternative presentation.

Access is through the web browser, or programmatically via the RESTful interface in XML or in Resource Description Framework. External resources can be searched via the FAIRDOMHub interface through available RESTful Application Programming Interfaces (APIs). For example, the BioModels database (5) can be searched together with the models in FAIRDOMHub, allowing users to place results in a broader context. The main web user interface allows users to browse the *Yellow Pages* (registered programmes, projects, institutions and people), the *Experiments* (Investigations, Studies and Assays), the *Research Assets* (Data, Models, Standard Operating Procedures, BioSamples, Organisms and Publications) and *Activities* (presentations and events). Rich browsing enables navigation between and across these categories and a graphical representation shows how individual assets are related to Assays, Studies and Investigations. The interface is designed to be interactive with the user navigating through the graph. Figure 1 shows an *Investigation*, one of its constituent Studies, Assays in that study, and a selection of associated research assets. For each uploaded asset, its version, license and access activity are recorded. If assets are published they are open to users without an account. Others are restricted through a comprehensive permissions system that controls visibility to projects, subgroups and even individuals. In some cases, metadata descriptions are visible, but the content is only available after contact with the owners, via the *Request Data File* link. Registered users can subscribe to specific assets to be notified of changes. Content is indexed on upload and available through a Solr search. Metadata from assets uploaded in JERM-compliant formats is extracted and stored as semantic annotations to assist navigation and search. To compare models and identify potential overlaps, semantic annotations are used to link reaction species to their exact definitions and structures in public databases such as BRENDA, SABIO-RK and ChEBI.

### Curation and review

The FAIRDOMHub is a public repository with an open submissions policy. New registrations from consortia are activated by a FAIRDOM administrator and then researchers administer their own programmes and projects, assigning membership and project roles. FAIRDOMHub provides curated, standards-compliant templates for data entry and standard recommended formats for models. The majority of assets are shared publicly only when research papers have been accepted. Potential re-users can therefore assume that the data sets and/or models have been peer-reviewed, but not necessarily that their formats and metadata contents have been curated. The FAIRDOM project offers curation services to consortia with direct FAIRDOM project support, including consultations for data structure and formats and model standards compliance. Technical curation of mathematical models published in a number of journals (i.e. *FEBSJ, Microbiology, Metabolomics, IET Systems Biology*) is also provided via the link to JWS Online. Secure access for pre-publication peer review is supported.

### Publishing

FAIRDOMHub aims to help researchers share systems biology results along with relevant data, models and protocols to promote reuse and support reproducible publication. For example, support for the SED-ML format, combined with links to the model and publication in the ISA structure, generates a reproducible record of the model simulation events for figures in published articles. Sharing and publishing of individual and collections of assets is governed by assigned credentials; for example sharing permissions can be changed by anyone granted *manage* privileges by the original uploader. ‘One-click publishing’ enables an asset and selected associated assets to be bundled into a ‘snapshot’ and assigned a DOI. Snapshot collections can be whole *Investigations* or smaller collections of *studies* and *assays*. The snapshot DOI serves as a supplementary material link in journals. Snapshots can also be packaged into zip files and exported. The snapshots are a form of Research Object (11); digital collections of resources packaged together with a citable identifier and enriched by metadata describing the collection, and relationships between components. For interoperability the Research Object and the COMBINE Archive specifications have been aligned.

### Versioning

For each uploaded asset, its version is recorded; as can relationships indicating if it was derived from, or influenced by, another asset. The version number is displayed with each entry and a comments field allows data uploaders to describe what has changed between versions. The snapshot DOI for publication fixes the version of the assets to be those reported in the paper. Of course assets may continue to be developed, in particular models; users can therefore navigate to subsequent versions through FAIRDOMHub. Plugins support model version visualization and comparison.

## DISCUSSION

The FAIR guiding principles of data stewardship (13) promote data sharing and reuse. FAIR adherence is becoming central to several Research Infrastructures within Europe such as ELIXIR (http://www.elixir-europe.org) and research funding data mandates increasingly require projects to make their data and models FAIR. Due to the heterogeneous and complex nature of systems biology, researchers in this area need more support to achieve FAIR data compliance. The FAIRDOMHub provides this functionality. It allows systems biologists to share and disseminate their research in a FAIR way that enables experimental data and mathematical modeling results to be presented and interpreted together. It has attracted large national and international consortia and small research groups as users. In addition the FAIRDOM website (http://www.fair-dom.org) provides a community ‘knowledge hub’ for data and model management issues, including links to community repositories, standards and initiatives, specialized software, webinars and tutorials and community groups.
